# Supplementary figures and images for: Pore-C sequencing identifies episome-driven chromosome conformation perturbations differentiating pneumococcal epigenetic variants
Source: PLoS Pathog. 2025 Aug 14;21(8):e1013392. doi: 10.1371/journal.ppat.1013392 (PMC12416852; doi:10.1371/journal.ppat.1013392)

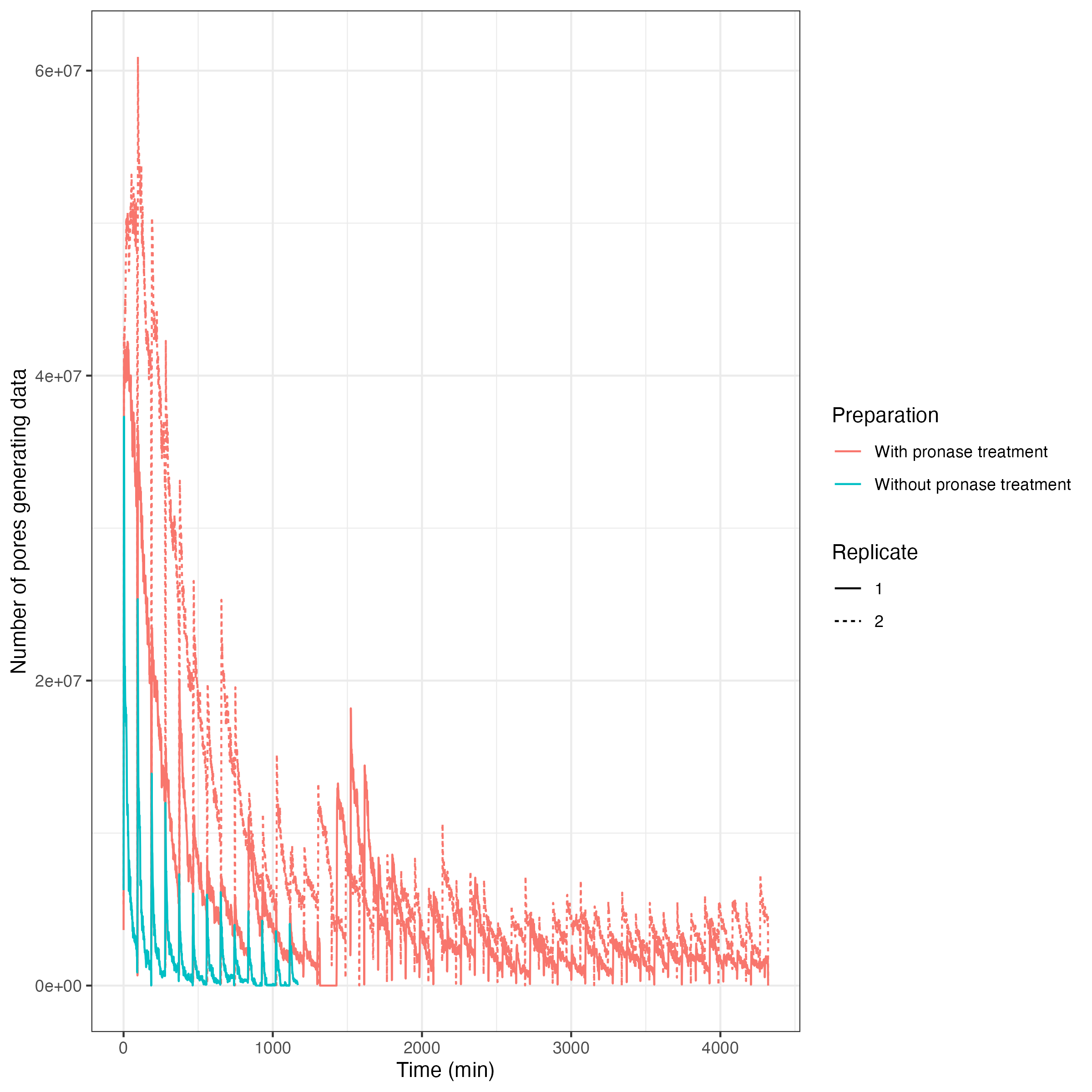

Supplement: S1 Fig — The line plots show the number of nanopores on a flow cell generating sequence data over the course of three sequencing runs. This declined over time as pores became blocked by DNA that remained cross-linked to proteins. The MinION device attempts to clear such blockages through regular transient voltage reversals, resulting in the jagged appearance of the curve. The lines are coloured according to whether the sequencing library was prepared using de-cross-linking with proteinase K only (one replicate), or proteinase K and pronase (two replicates). This demonstrated the additional pronase treatment substantially reduced the rate of pore blocking, resulting in an increased yield of sequence data. (TIF) [file ppat.1013392.s001.tif]

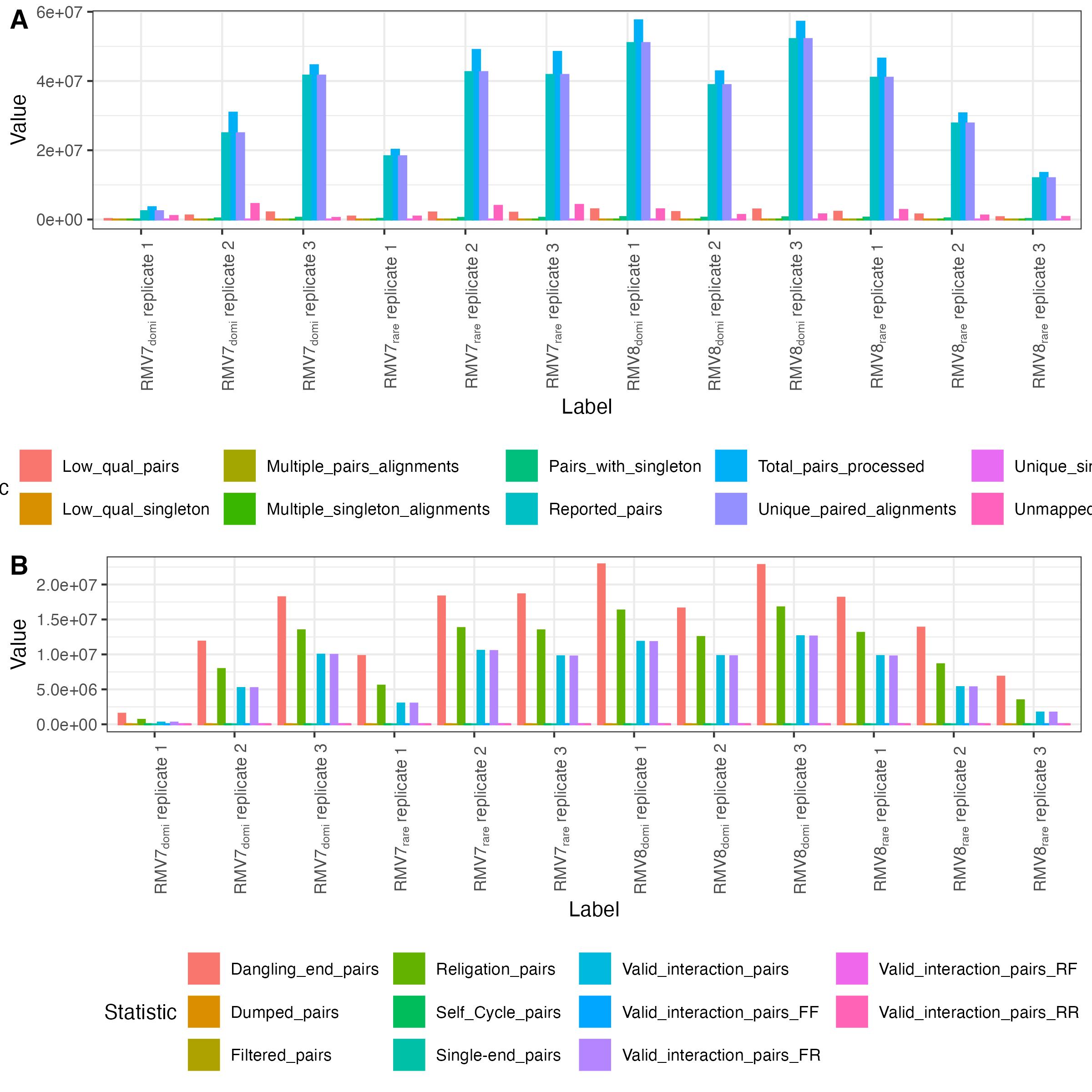

Supplement: S2 Fig — (A) Analysis of paired read alignment to the reference genome. This shows the majority of processed pairs were successfully mapped to the genome as unique, paired alignments. (B) Analysis of the aligned pairs. Most reads were split between three categories. The “dangling end pairs” correspond to reads from the ends of the same restriction fragment, which represents a failure to ligate a DNA molecule into a longer concatemer. The “relegation pairs” correspond to either to DNA molecules that were not digested, or neighbouring fragments that were ligated back together into their original form. The “valid interaction pairs” correspond to pairs that allow for a digestion and relegation to be inferred. A high proportion of these read pairs have the forward-reverse relative orientation expected of undigested DNA. (TIF) [file ppat.1013392.s002.tif]

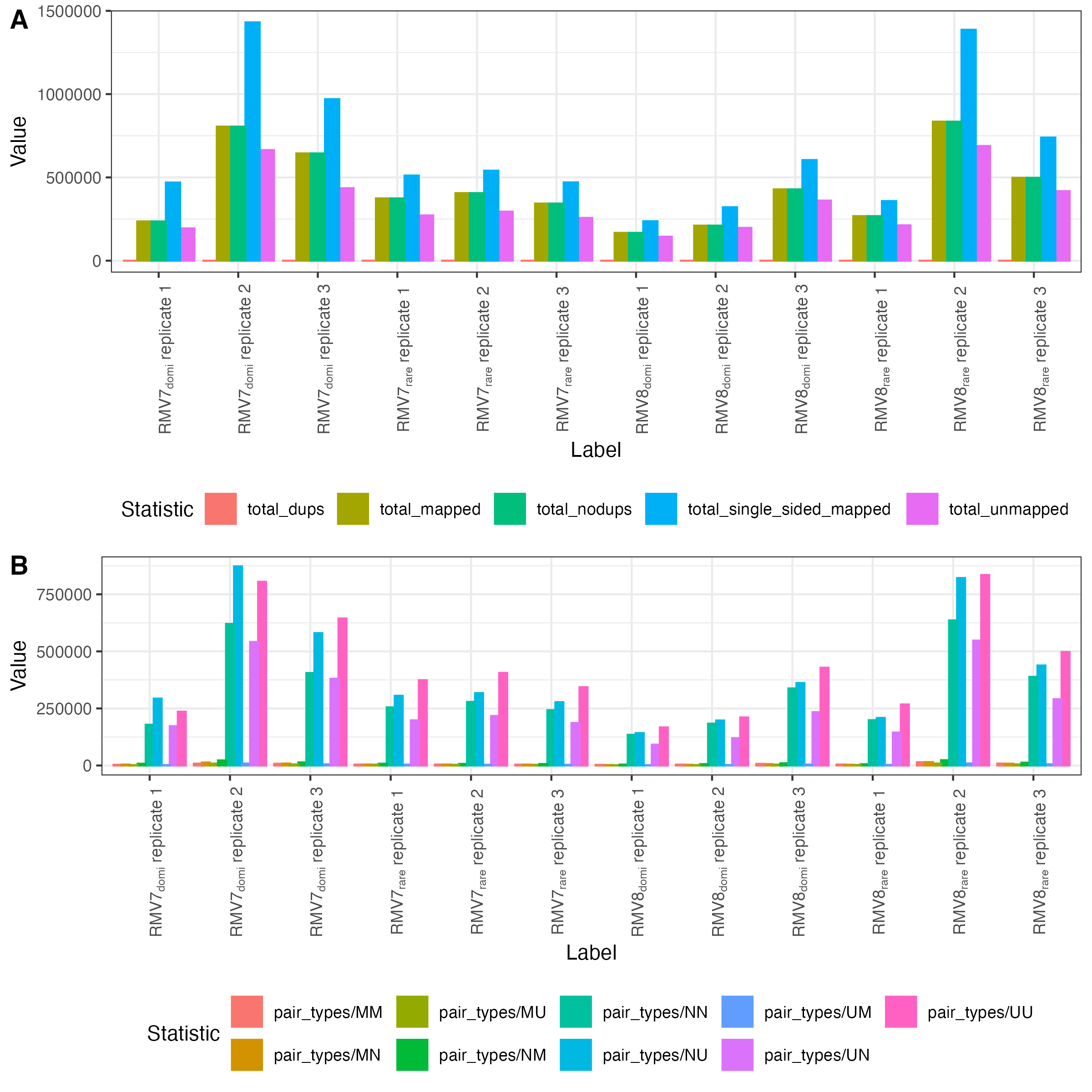

Supplement: S3 Fig — (A) Processing of sequence reads, split into pairs of restriction fragments. The modal categories comprised pairs in which only one of the two sequences could be mapped to the reference genome. Nevertheless, there were more pairs of sequences that could both be mapped to the reference than pairs of sequences in which neither fragment mapped to the reference. (B) Details of read mapping. All pairs were classified by the mapping status of the constituent reads. The unmapped reads from panel (A) were divided between categories in which at least one read was not able to be mapped to the reference genome (N), or mapped to multiple sites (M). The single-sided mapped reads were split into the four categories in which only one read could be uniquely mapped (U).Yet many read pairs consisted of paired fragments that were both uniquely mapped (UU), enabling inference of contacts across the genome. (TIF) [file ppat.1013392.s003.tif]

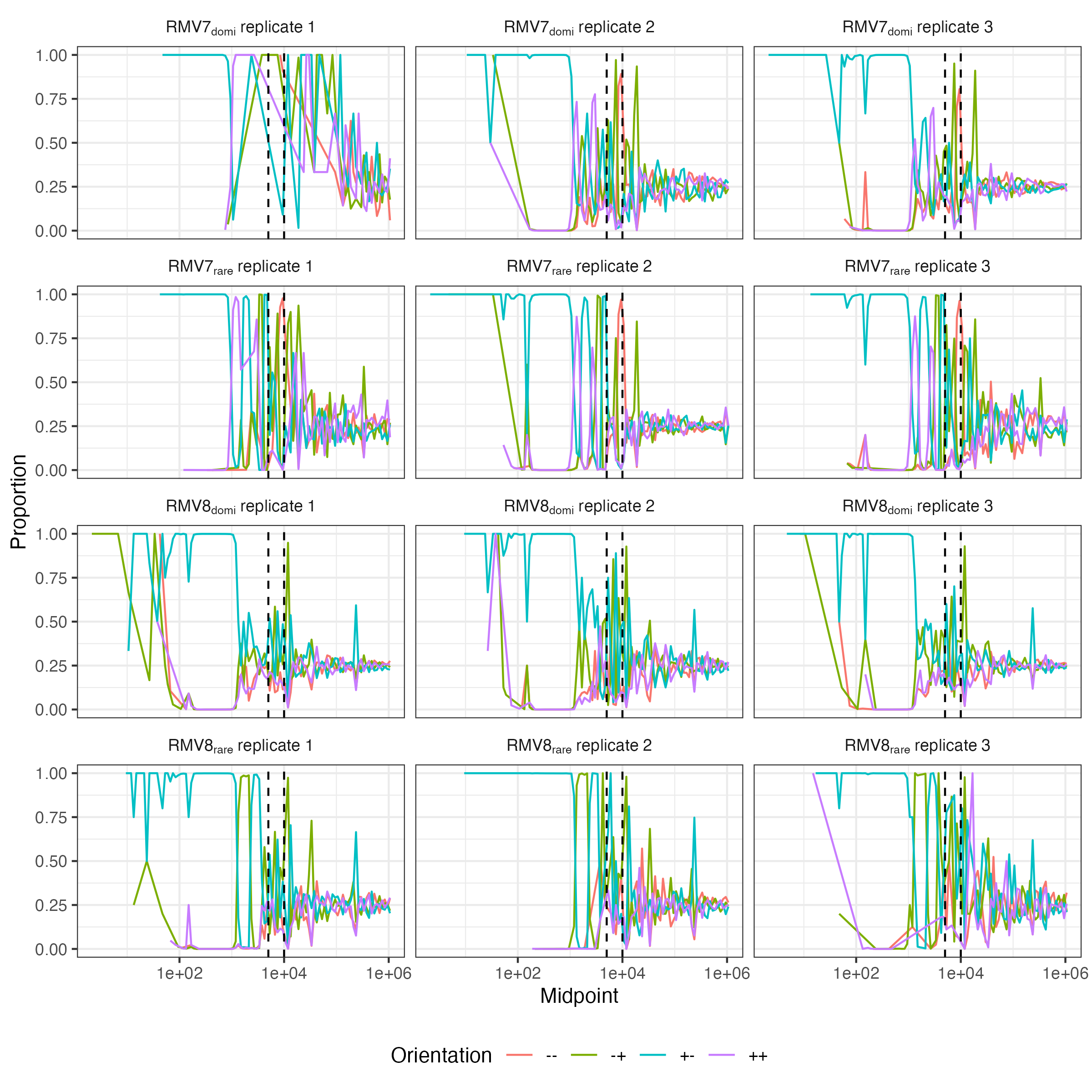

Supplement: S4 Fig — The read pairs were categorised into 100 bins, based on the distribution of logarithmically-scaled distances between the mapping locations of the pair members. For each bin, the proportions of read pairs mapping in the four possible orientations (both to the positive strand; both to the negative strand; the forward read mapping to the positive strand and the reverse read mapping to the negative strand; or the forward read mapping to the negative strand and the reverse read mapping to the positive strand) were calculated. The different orientations are represented by the colour of the lines. As Illumina read pairs are generated by sequencing initiated from each end of a DNA molecule, reads generated from undigested templates are expected to map to different strands of the genome (i.e., have a + /- or -/ + orientation). These orientations dominate over short distances, suggesting read pairs mapping a short distance from one another were generated from uncut genomic DNA. The dashed lines represent estimates of the convergence distance, at which digestion and religation is sufficiently frequent for the four orientations to each reach an approximately equal proportion of ~0.25. Each panel shows the data from an individual replicate. (TIF) [file ppat.1013392.s004.tif]

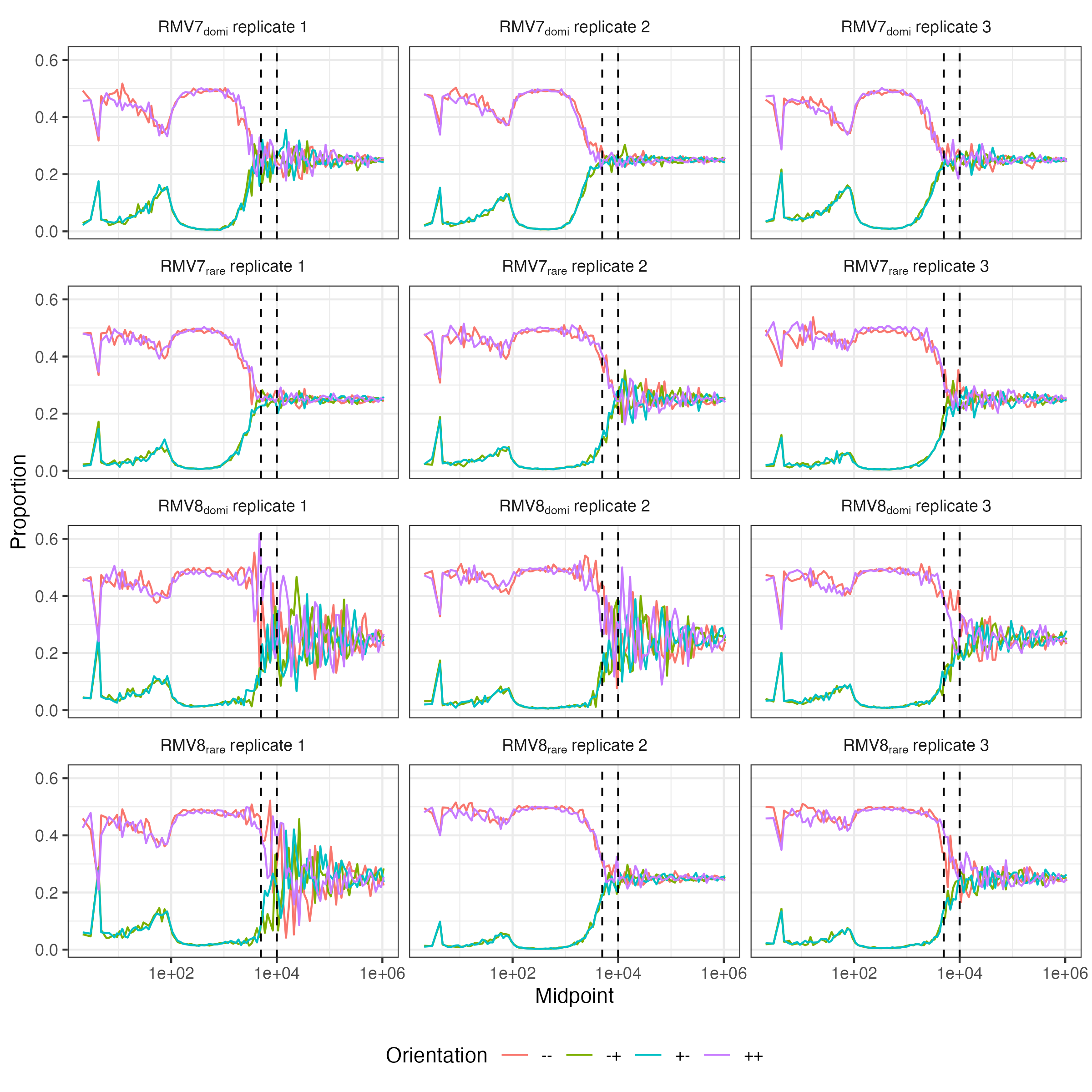

Supplement: S5 Fig — Data are shown as in S4 Fig. As Nanopore reads are generated as continuous sequence following initiation at one end of a molecule, data generated from an undigested template is expected to have a + /+ or -/- orientation. Therefore these orientations dominate over short distances, in contrast to the analyses shown in S4 Fig. (TIF) [file ppat.1013392.s005.tif]

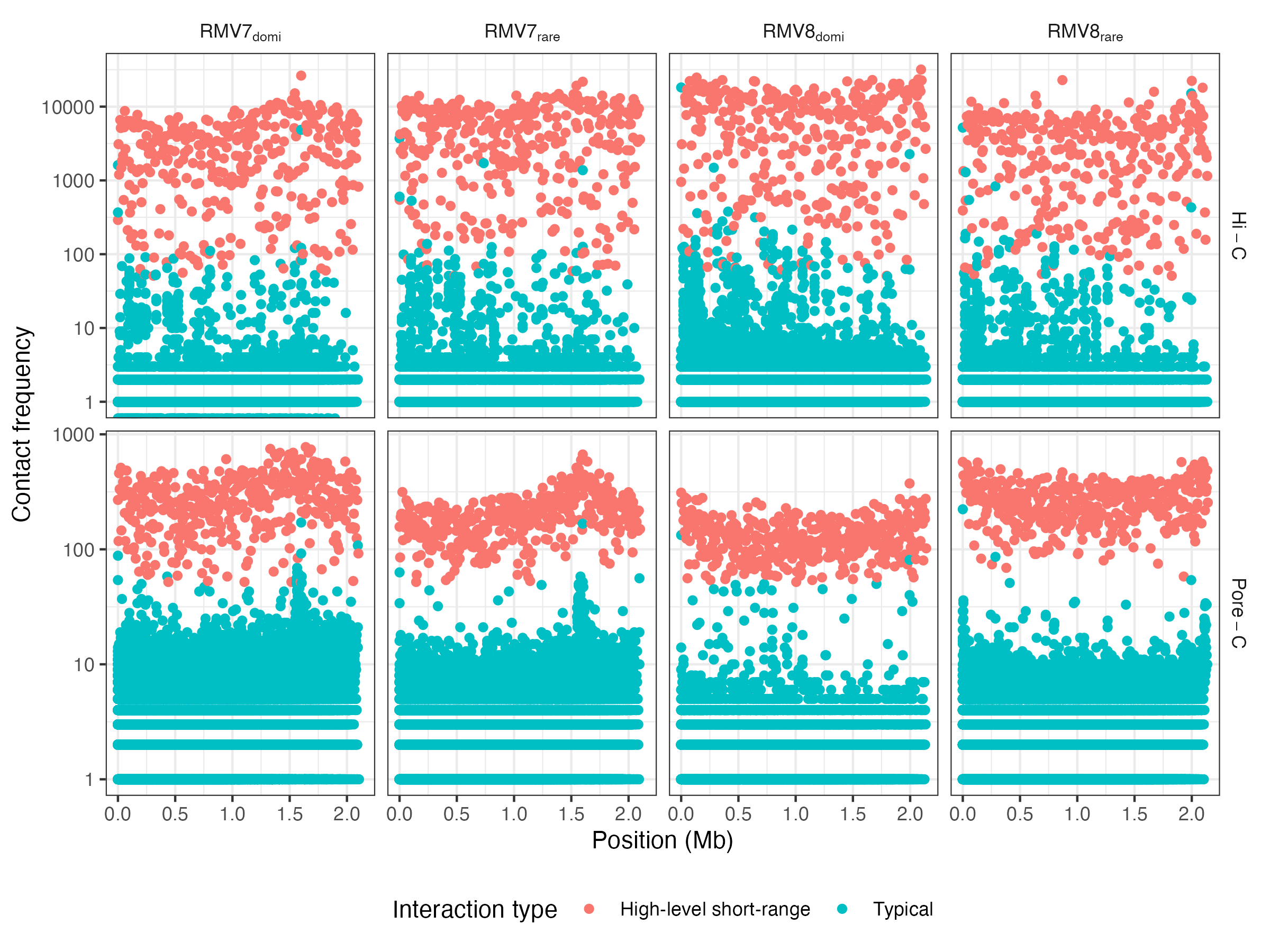

Supplement: S6 Fig — The absence of any spatial clustering of the short-range highly-interacting loci suggested they could not be explained by a process localised to a specific region, and therefore were most parsimoniously explained as artefactual products resulting from incomplete digestion of DNA. (TIF) [file ppat.1013392.s006.tif]

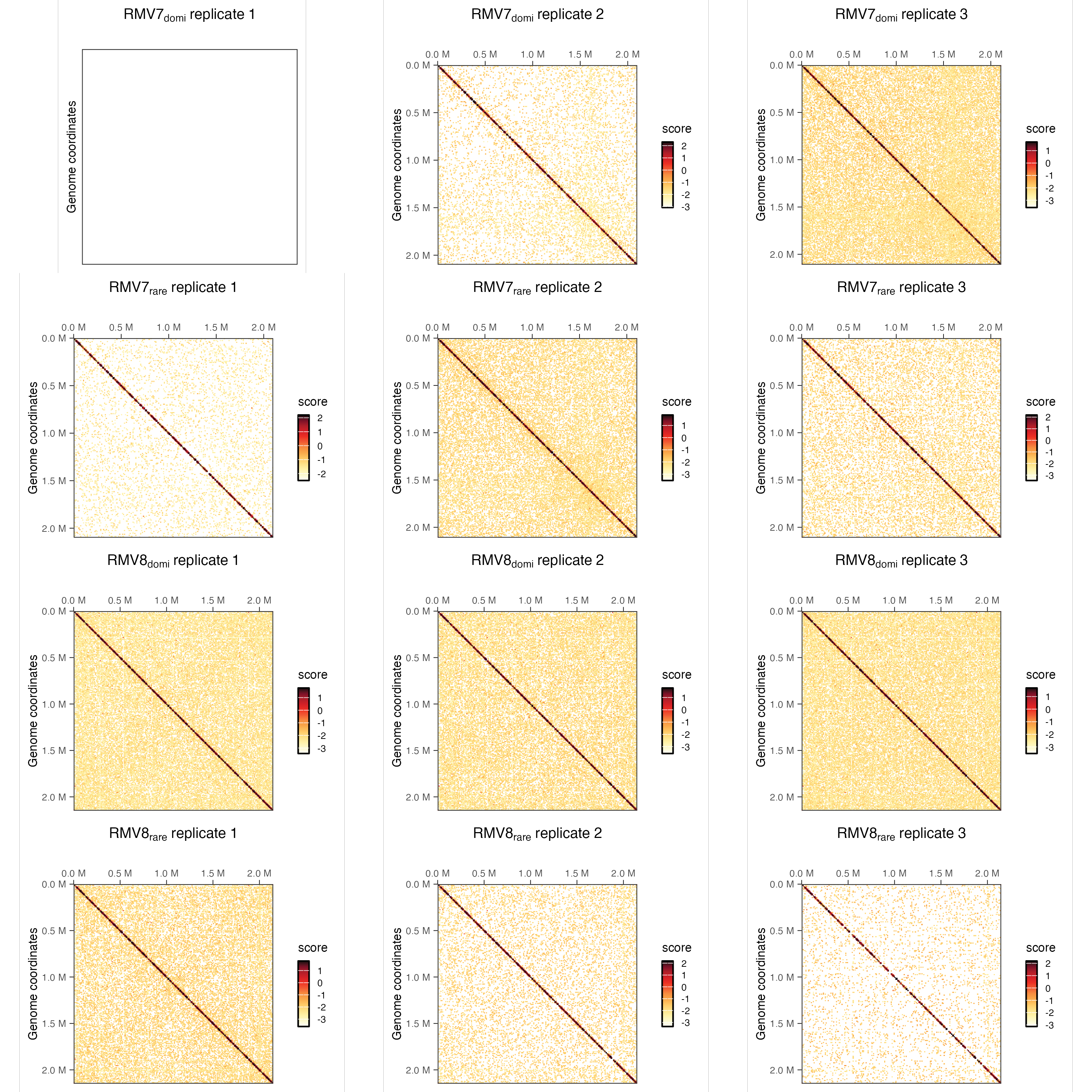

Supplement: S7 Fig — Insufficient long-range interactions were inferred from the first sample extracted from S. pneumoniae RMV7domi to generate a matrix. Each other matrix is symmetrical, with both the horizontal and vertical axes representing the length of the genome. Each cell is coloured to represent the frequency of interactions between the corresponding loci. Keys are provided for each replicate individually, to adjust the visualisation to the amount of data generated. (TIF) [file ppat.1013392.s007.tif]

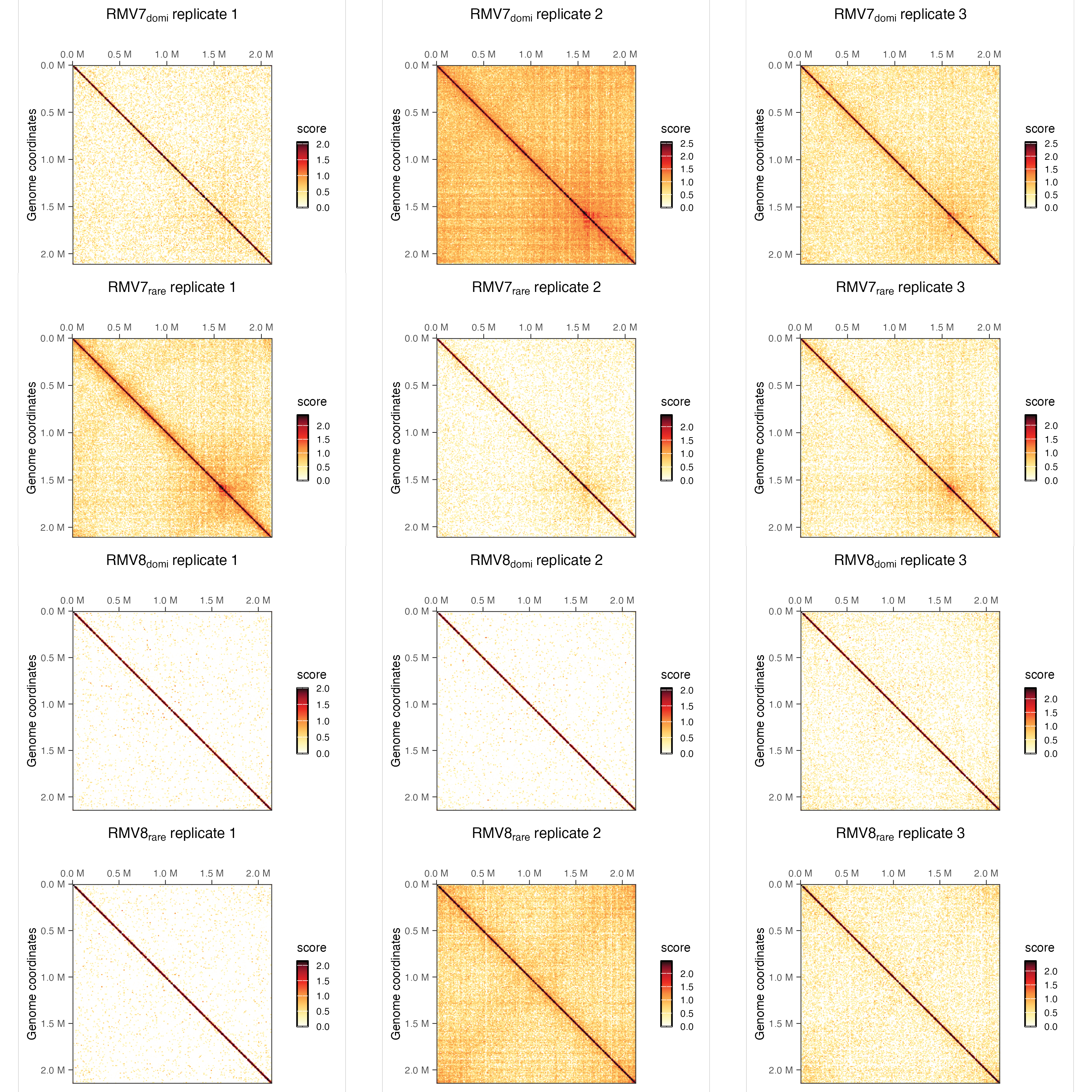

Supplement: S8 Fig — Data are shown as in S7 Fig. (TIF) [file ppat.1013392.s008.tif]

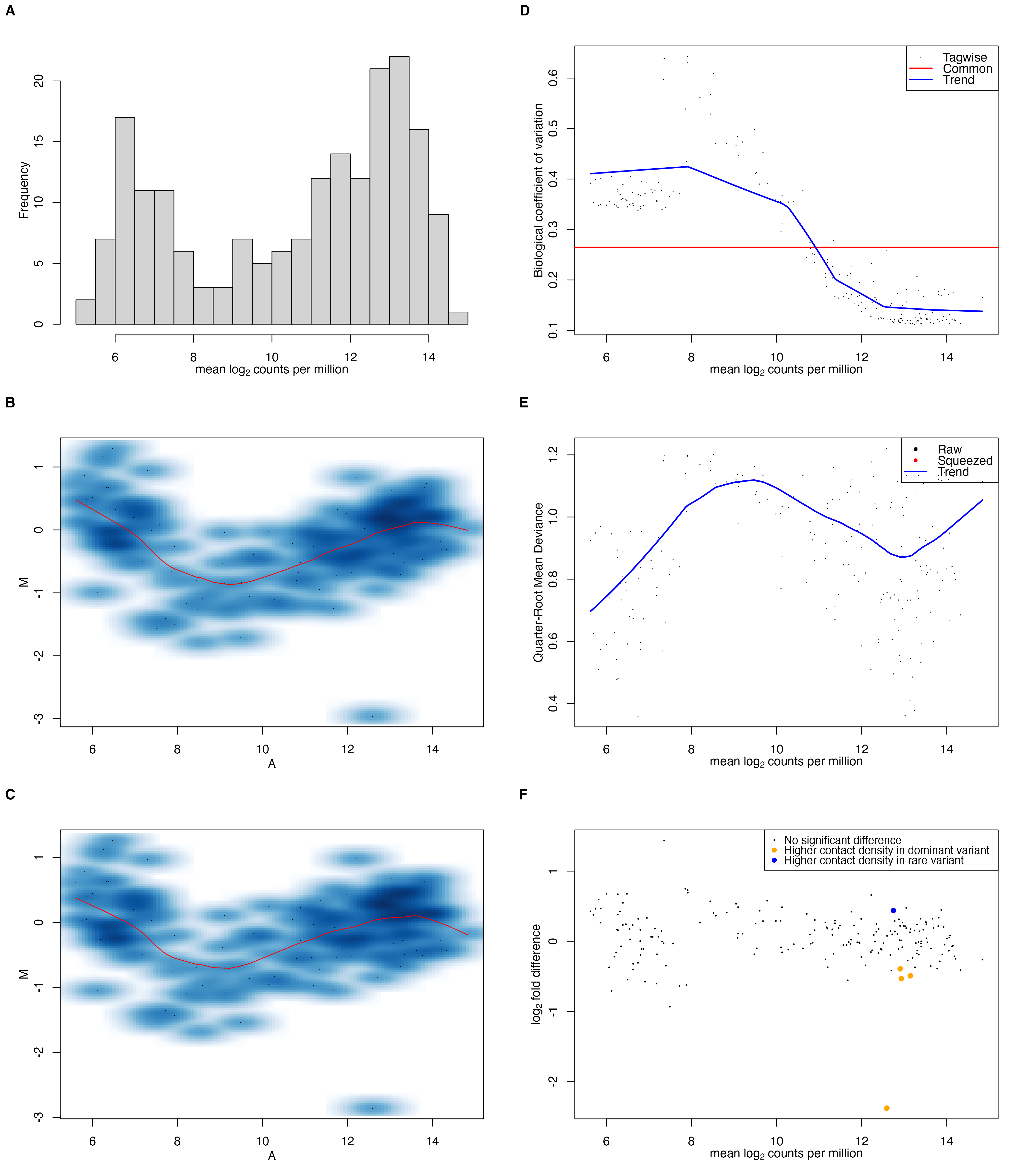

Supplement: S9 Fig — All analyses were conducted using a locus size of 10 kb. (A) Histogram showing the distribution of contact frequencies across all loci and replicates as mean log2 counts per million reads. (B) MA plot comparing the mean log2 counts per million reads (denoted A) against the ratio of log2 counts per million reads in the rare variant relative to the dominant variant (denoted M) for a representative pair of biological replicates. The density of points is represented by the blue shading. The LOESS regression line is shown in red. (C) MA plot of the same data after normalisation. (D) Relationship between the biological coefficient of variation, relating to the dispersion parameter of the negative binomial distribution used to fit the generalised linear model, and the mean log2 counts per million reads. (E) Relationship between the quasi-likelihood dispersion and the mean log2 counts per million reads estimated by fitting the generalised linear model to the contact frequency data. (F) MA plot of normalised data, highlighting loci found to have a significantly differing density of contacts between the two variants. (TIF) [file ppat.1013392.s009.tif]

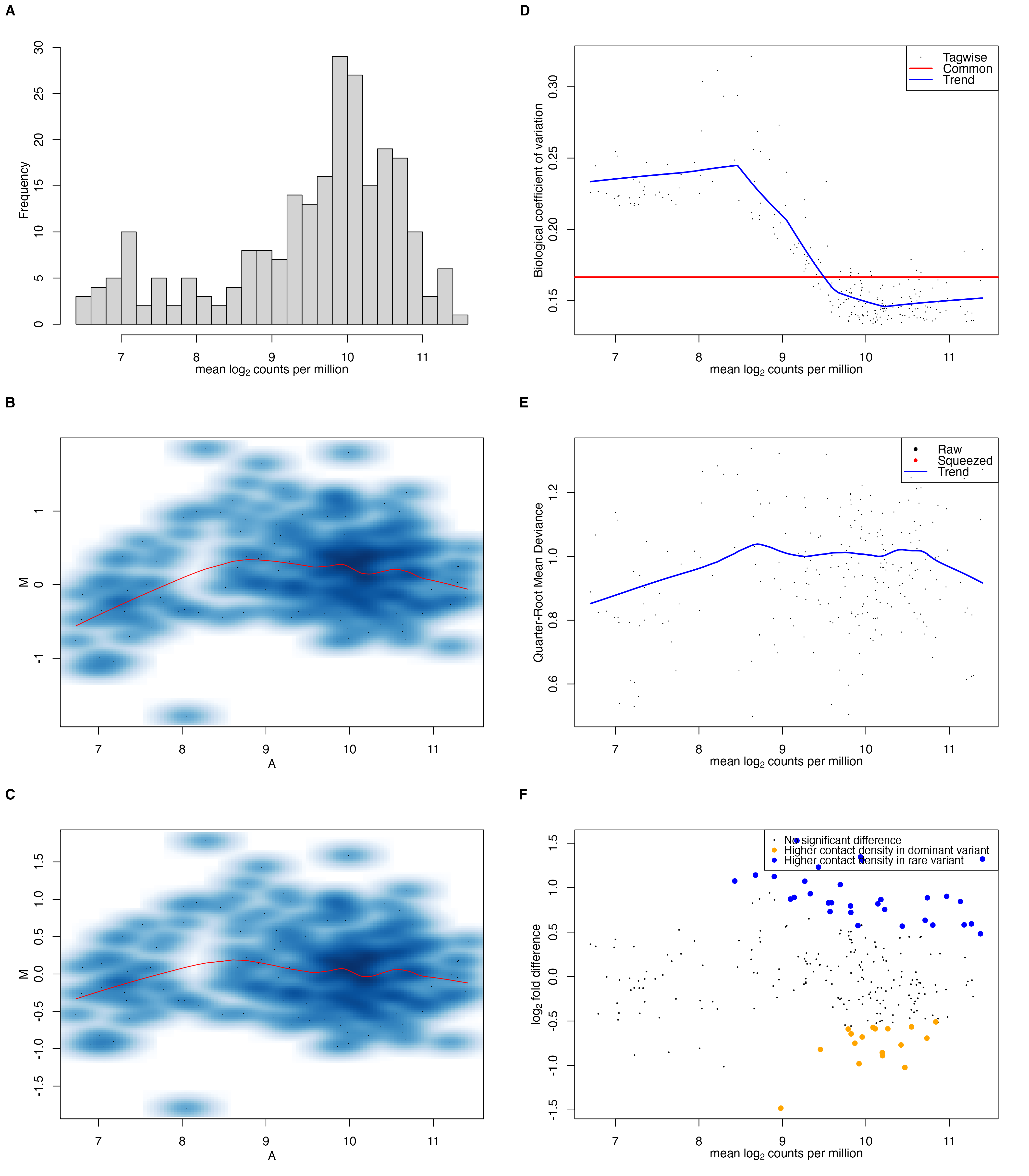

Supplement: S10 Fig — All analyses were conducted using a locus size of 10 kb. Data are shown as in S9 Fig. (TIF) [file ppat.1013392.s010.tif]

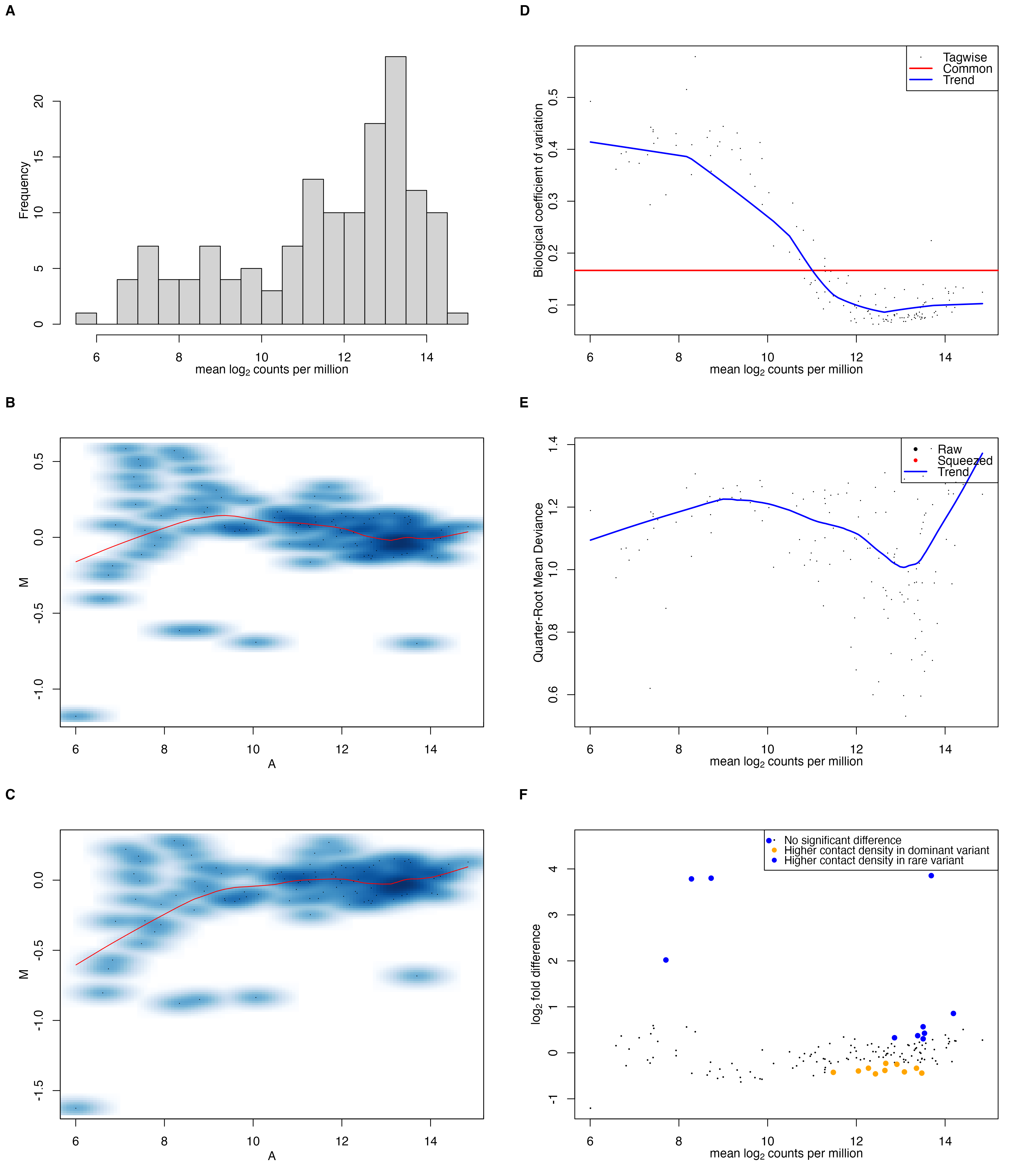

Supplement: S11 Fig — All analyses were conducted using a locus size of 10 kb. Data are shown as in S9 Fig. (TIF) [file ppat.1013392.s011.tif]

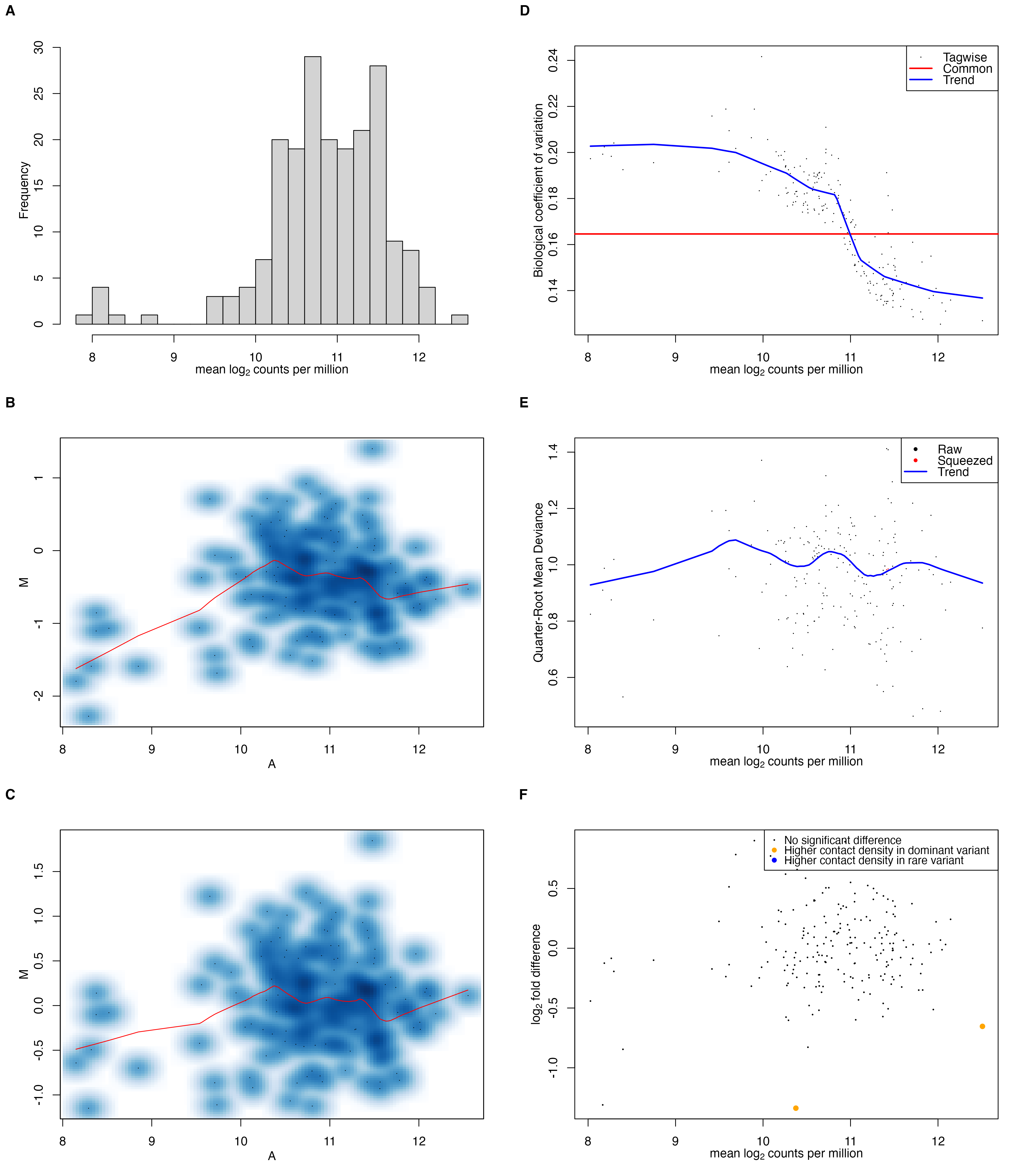

Supplement: S12 Fig — All analyses were conducted using a locus size of 10 kb. Data are shown as in S9 Fig. (TIF) [file ppat.1013392.s012.tif]

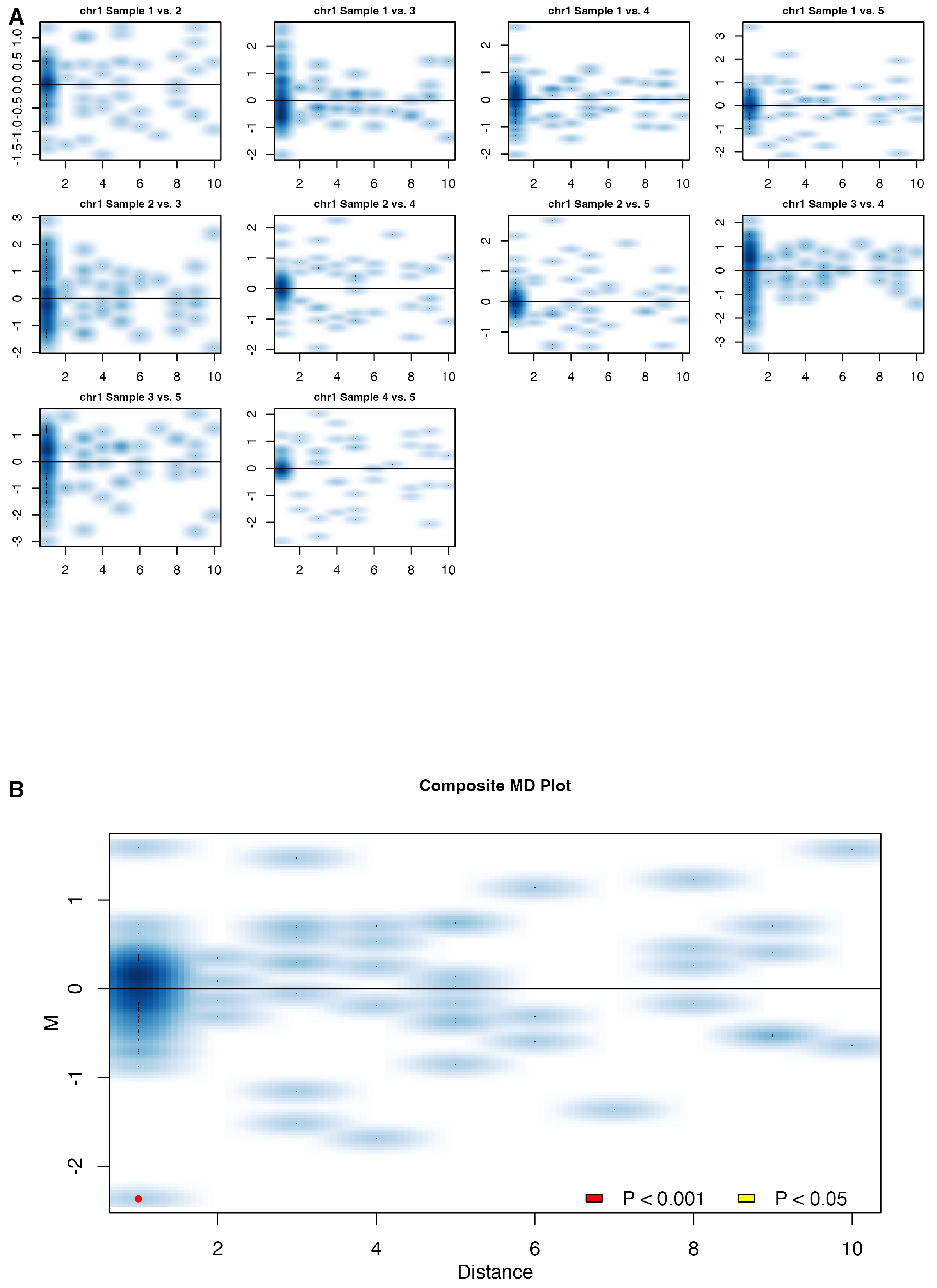

Supplement: S13 Fig — All analyses were conducted using a locus size of 10 kb. (A) MD plots for each pairwise comparison between replicates after joint normalisation by a cyclic LOESS process. Each plot shows the ratio of log2 counts per million reads in one replicate relative to another (denoted M), relative to the distance between the interacting loci (denoted D), enumerated as the difference between the indices of the loci. (B) Composite MD plot summarising the output of the test for differential contact intensities across all replicates. The position of points on the vertical axis represents the log2 fold difference between the variants, with points higher on the axis when the contact density is higher in the rare variant relative to the dominant variant. Points are coloured according to the categorisation of their false discovery rate (i.e., q value), calculated after a Benjamini-Hochberg correction for multiple testing. (TIF) [file ppat.1013392.s013.tif]

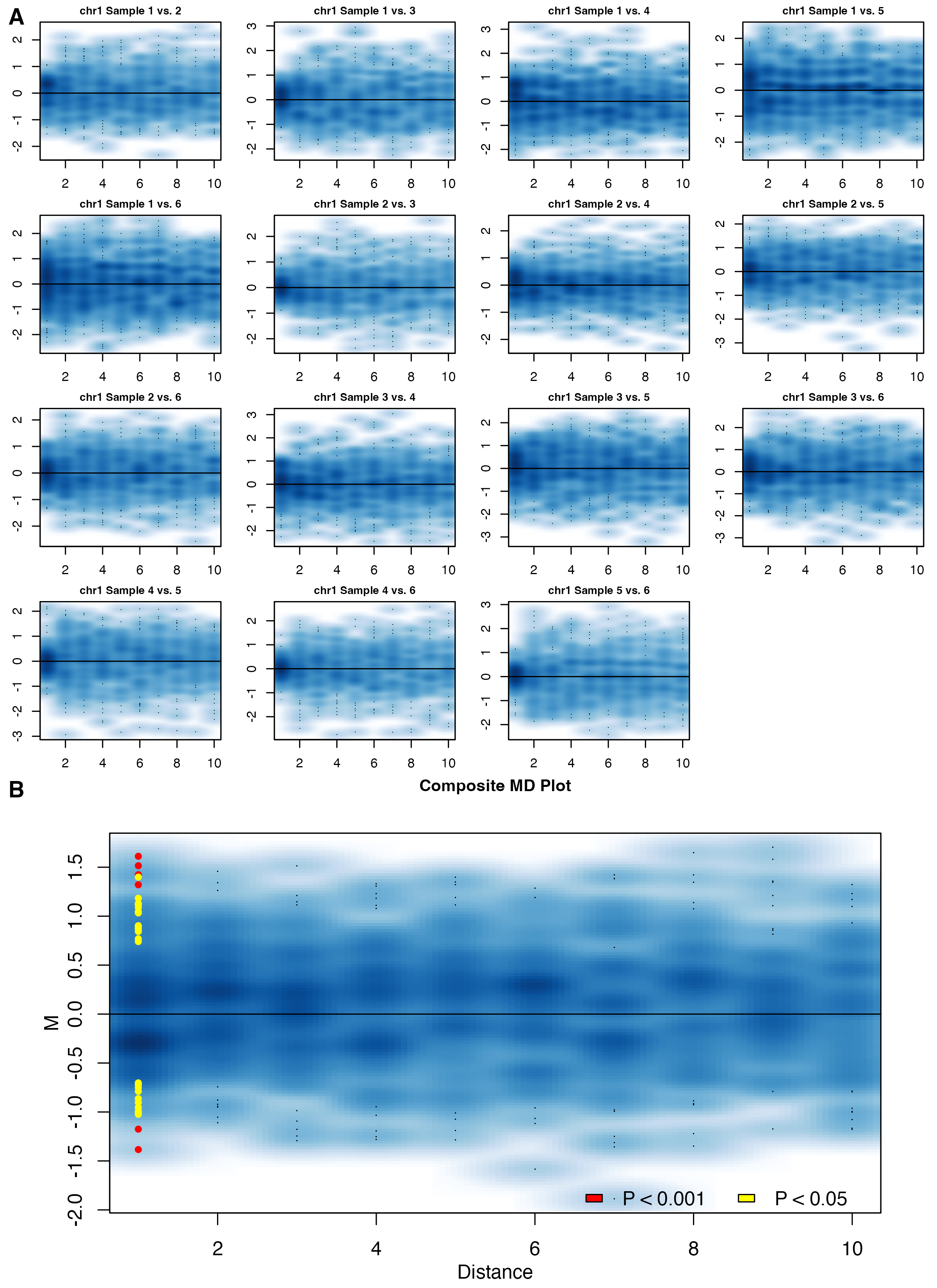

Supplement: S14 Fig — All analyses were conducted using a locus size of 10 kb. Data are shown as in S13 Fig. (TIF) [file ppat.1013392.s014.tif]

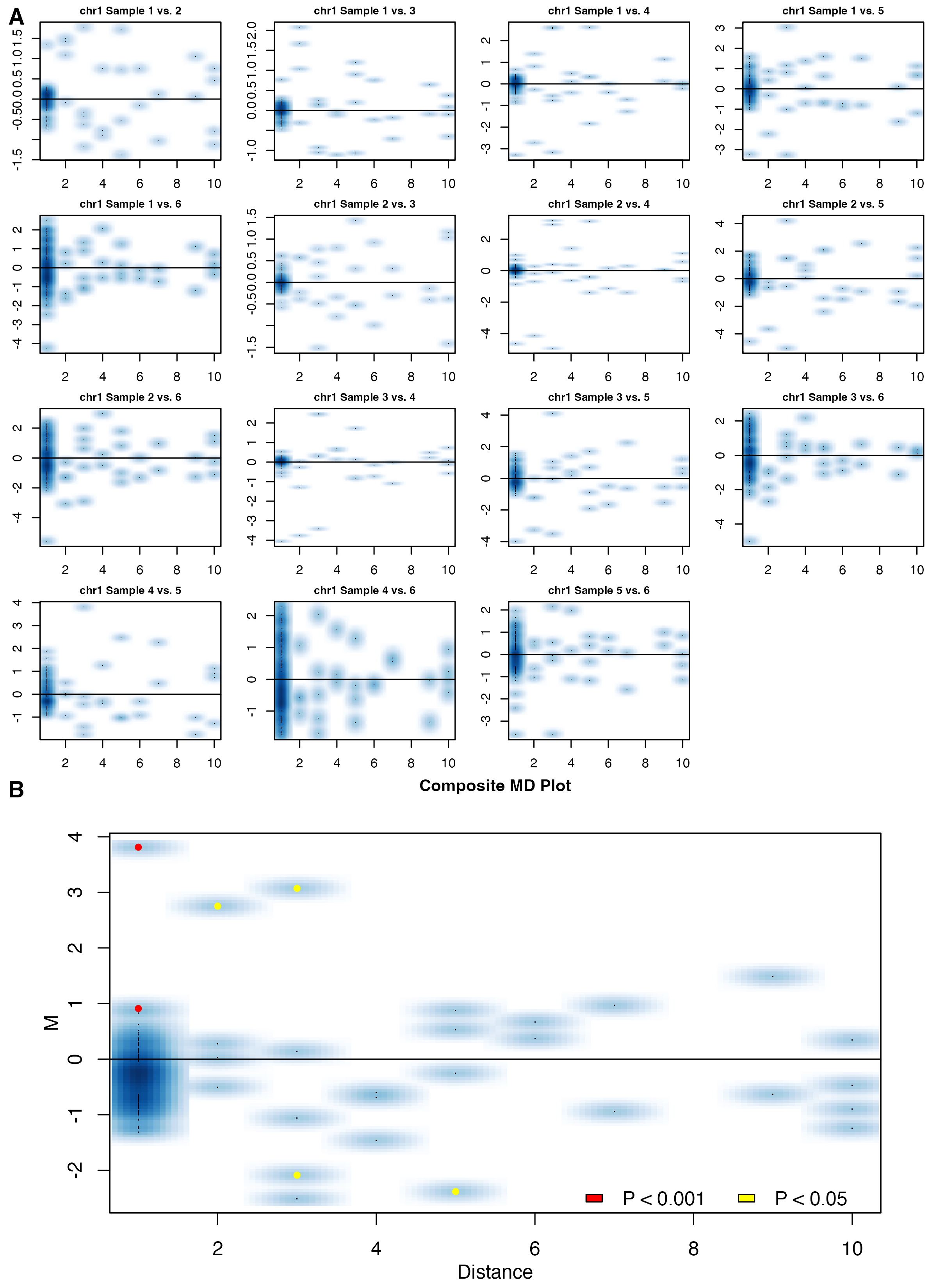

Supplement: S15 Fig — All analyses were conducted using a locus size of 10 kb. Data are shown as in S13 Fig. (TIF) [file ppat.1013392.s015.tif]

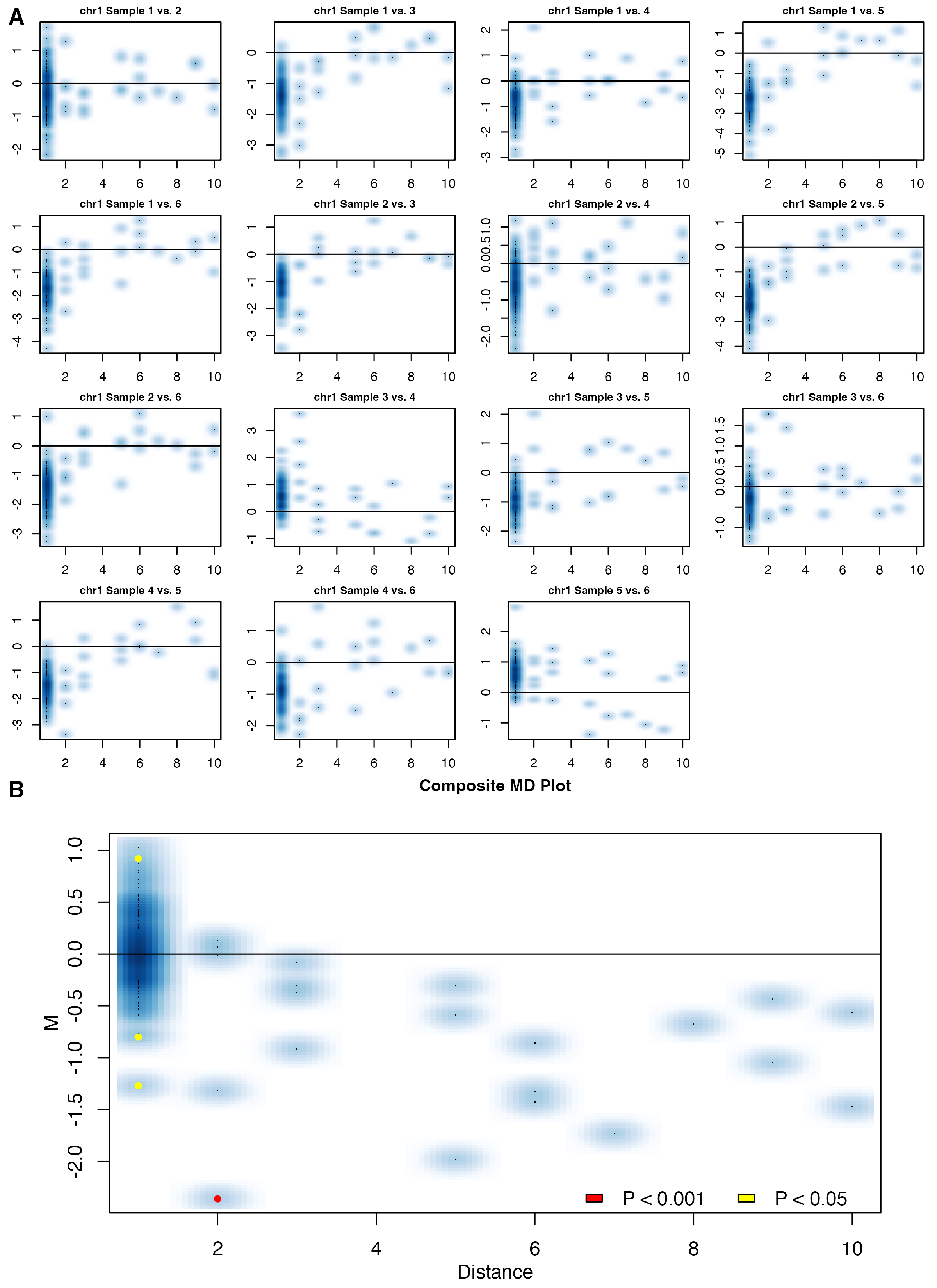

Supplement: S16 Fig — All analyses were conducted using a locus size of 10 kb. Data are shown as in S13 Fig. (TIF) [file ppat.1013392.s016.tif]

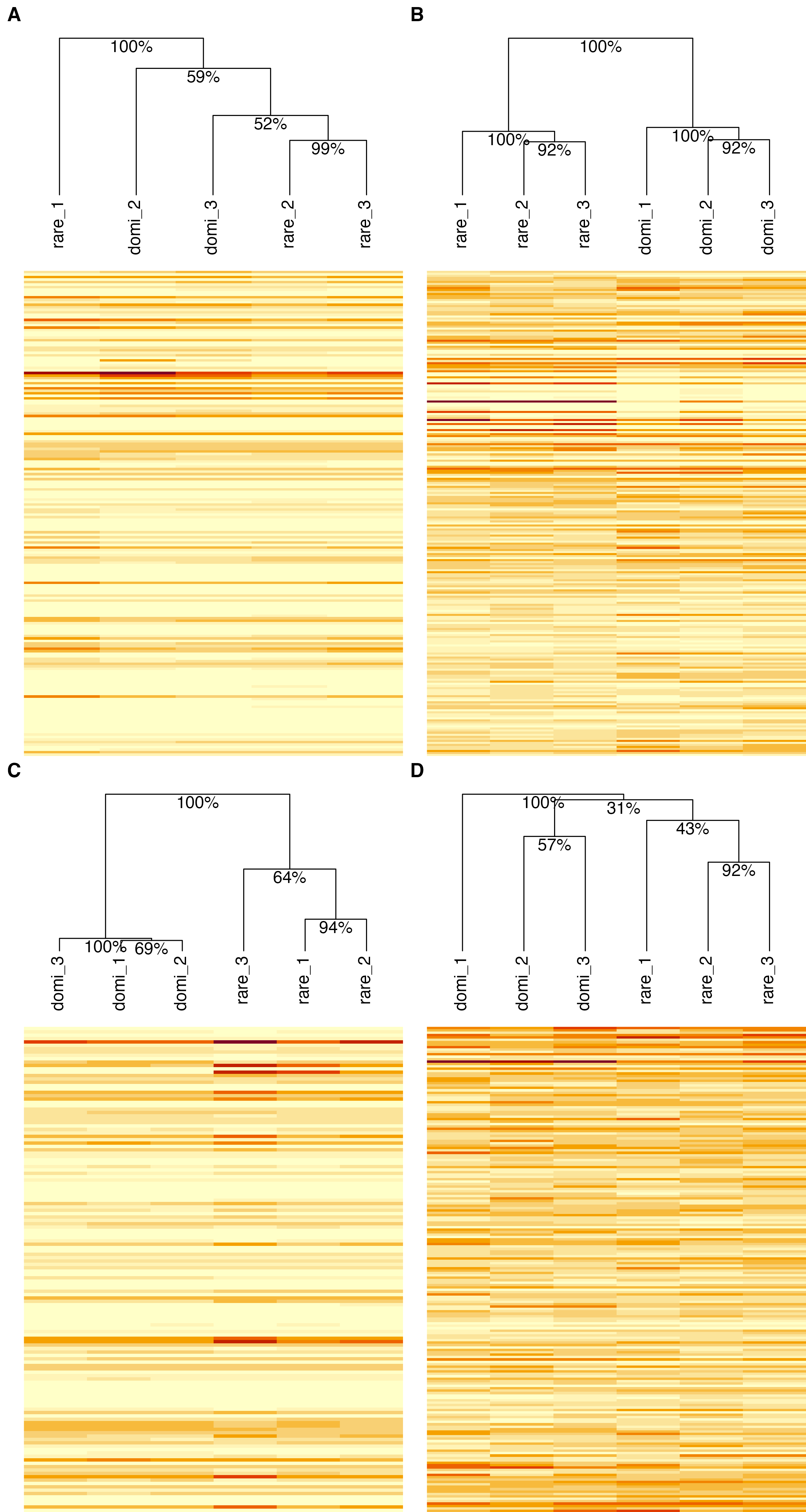

Supplement: S17 Fig — In each heatmap, each column is a different replicate dataset from either the dominant (domi) or rare variant. Each row represents a locus. The colour of each cell represents the intensity of contacts at a locus in a replicate following normalisation as part of the diffHiC analyses. The variants are clustered based on their similarity, as shown by the dendrograms. The nodes of the dendrogram are labelled with the corresponding bootstrap support values, expressed as percentages. The panels show (A) Illumina Hi-C data for RMV7 (B) Pore-C data for RMV7 (C) Illumina Hi-C data for RMV8 (D) Pore-C data for RMV8. (TIF) [file ppat.1013392.s017.tif]

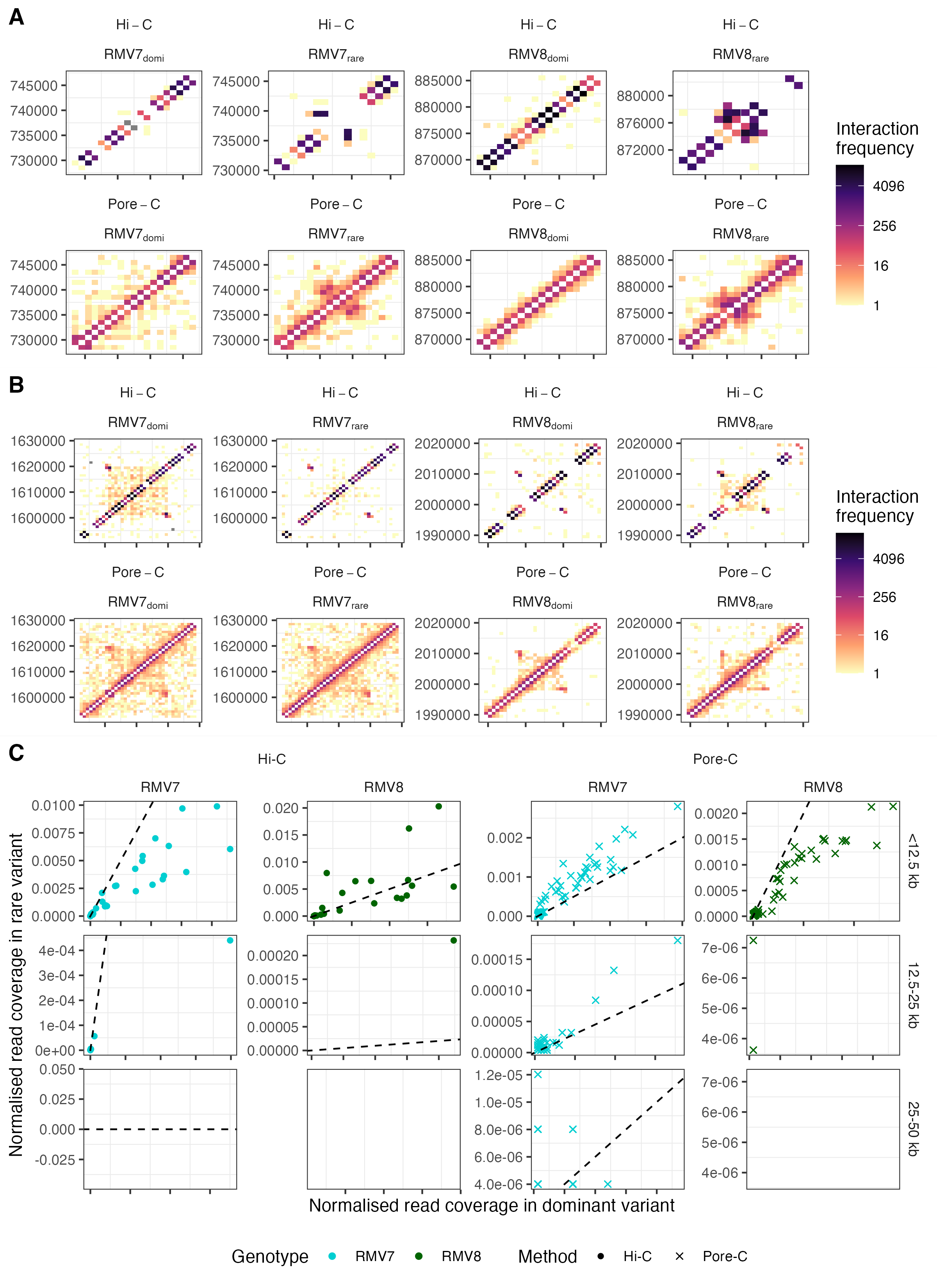

Supplement: S18 Fig — (A) Contact frequency matrices at the tvr loci of RMV7 and RMV8 at a resolution of 1 kb. Data are shown as in Fig 1D. Each rare variant dataset shows distinctive off-diagonal contacts that are diagnostic of rearrangements within the tvr loci, relative to the dominant variant reference genome. This confirms that the dominant and rare variant datasets have been correctly identified. (B) Contact frequency matrices within PRCIdnaN, for the RMV7 datasets, or within PRCImalA, for the RMV8 datasets, at a resolution of 1 kb. Data are shown as in Fig 1D. (C) Scatterplots comparing the contact frequencies within PRCIdnaN and PRCImalA between the epigenetically-distinct variants. The cumulative contact frequencies between each 1 kb locus were calculated across the three biological replicates for each combination of methodology and variant. These values were normalised by dividing them by the total number of contacts inferred in the corresponding datasets. These normalised contact frequencies were compared between the dominant and rare variants for the loci within PRCIdnaN, in RMV7, and PRCImalA, in RMV8. The comparisons were segregated by the distance between the interacting loci. The dashed lines are the lines of identity. These simple comparisons concur with the diffHiC and multiHiCcomparison analyses that found the Hi-C and Pore-C data implied opposing changes in the frequencies of contacts within these PRCIs between the pairs of variants. (TIF) [file ppat.1013392.s018.tif]

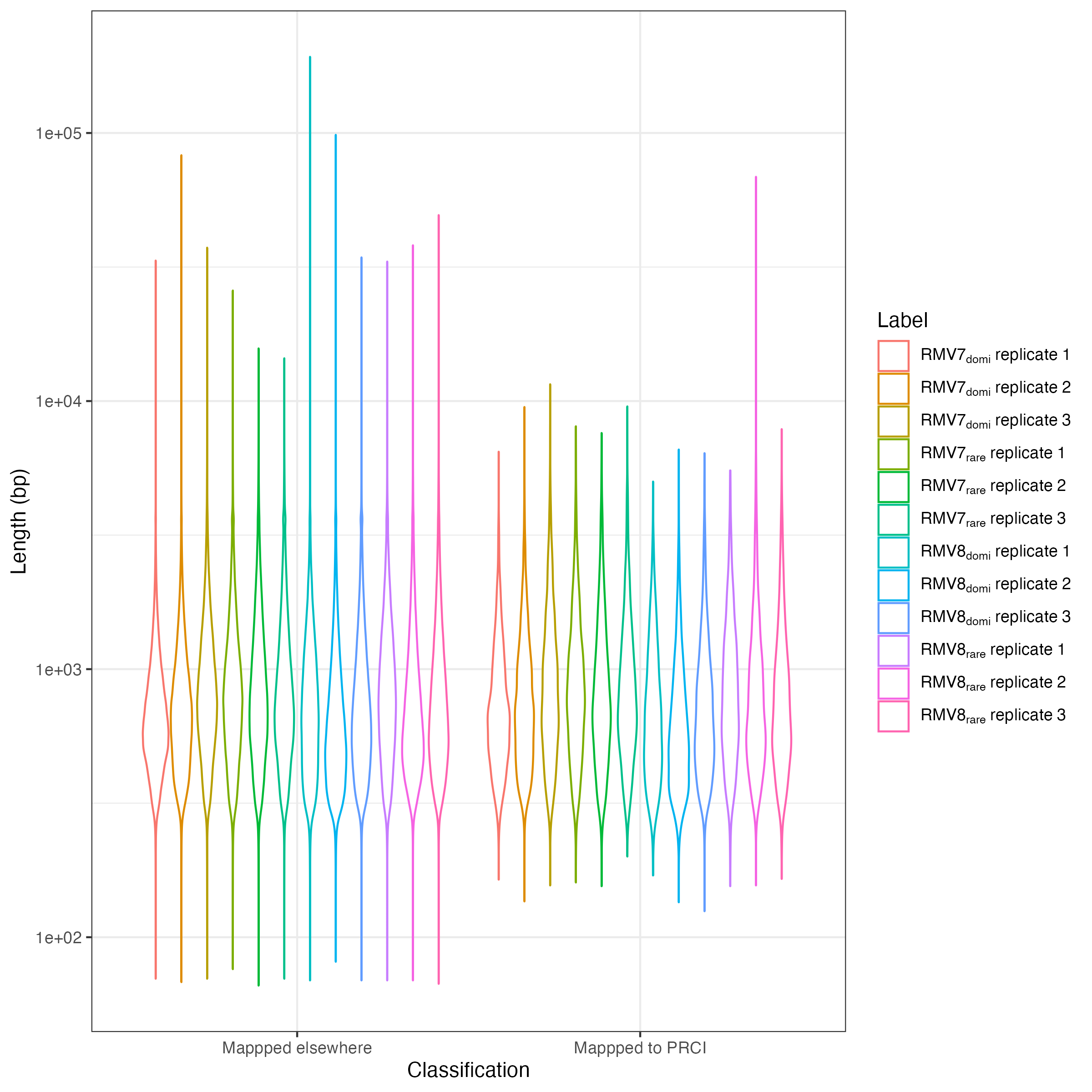

Supplement: S19 Fig — (TIF) [file ppat.1013392.s019.tif]

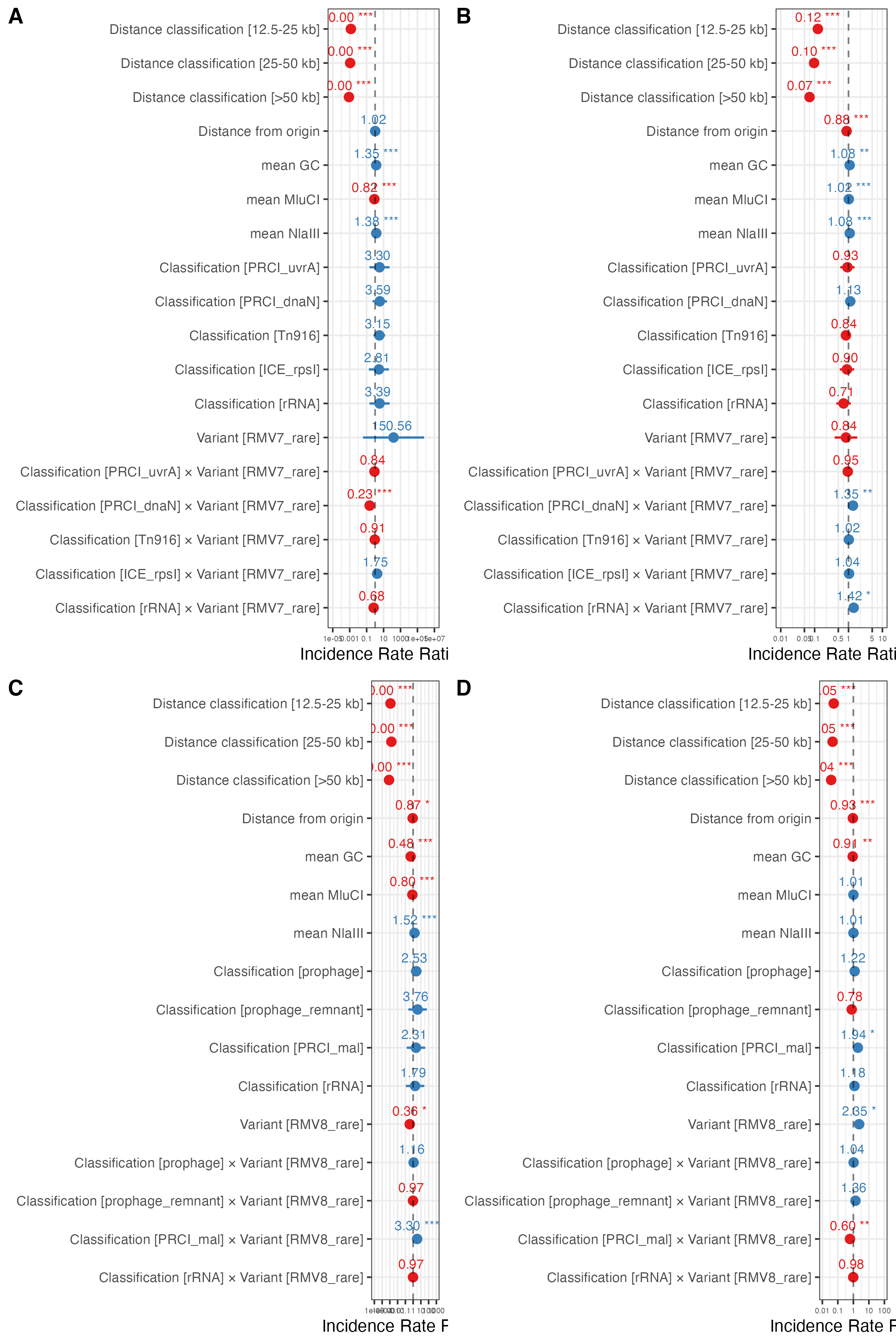

Supplement: S20 Fig — Points represent the maximum likelihood estimate of parameter values, and are coloured by whether they increase (blue) or decrease (red) contact frequencies. The error bars represent the 95% confidence intervals of the estimates. The asterisks indicate statistically significant deviations from one: * denotes p < 0.05; ** denotes p < 0.01, and *** denotes p < 0.001. (A) Model fit to the RMV7 Hi-C data. (B) Model fit to the RMV7 Pore-C data. (C) Model fit to the RMV8 Hi-C data. (D) Model fit to the RMV8 Pore-C data. (TIF) [file ppat.1013392.s020.tif]

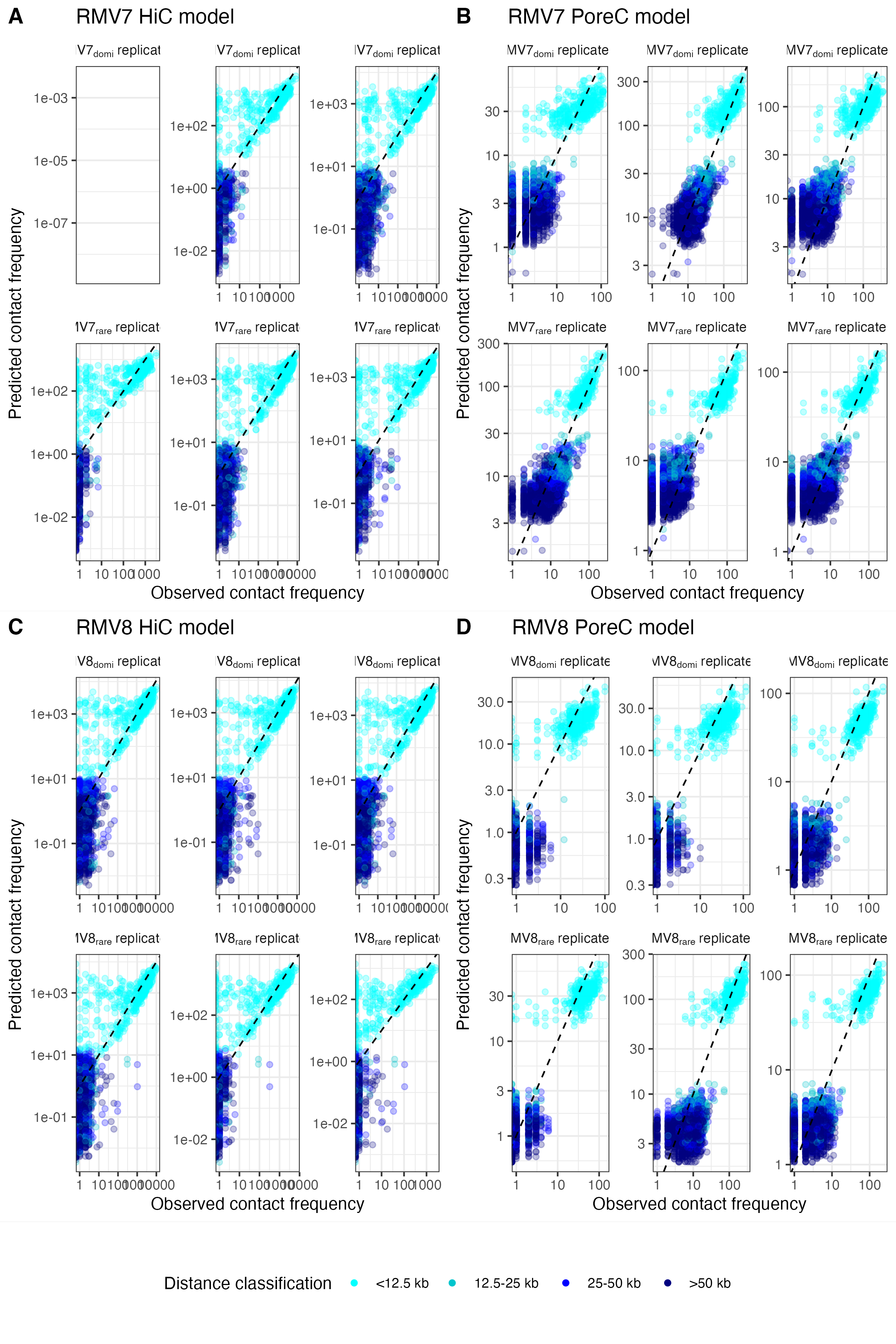

Supplement: S21 Fig — These plots, generated by the R package performance, test whether the model performs poorly in reproducing observed values, whether it is mis-specified, or whether there are difficulties in estimating individual parameter values. (TIF) [file ppat.1013392.s021.tif]

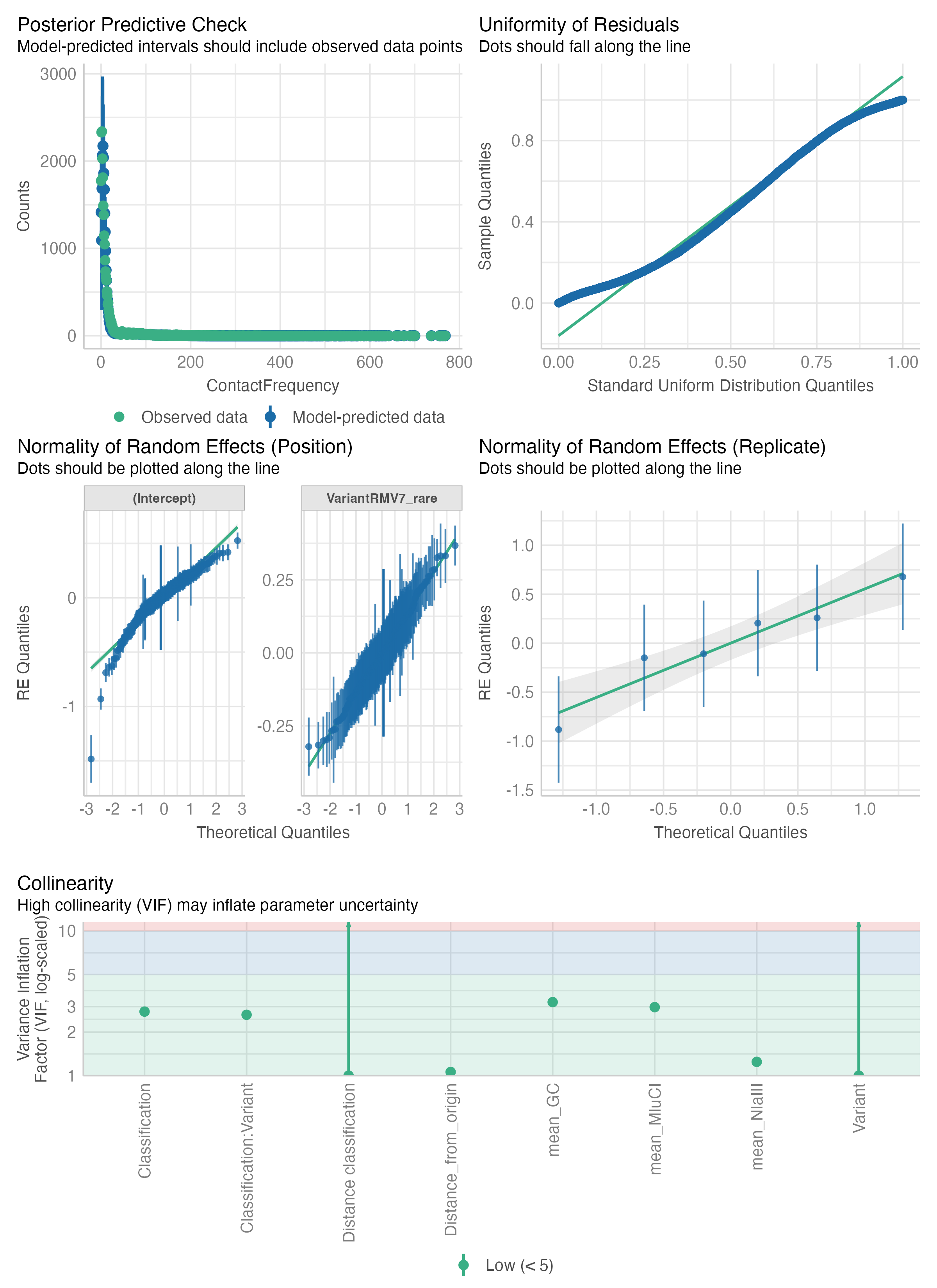

Supplement: S22 Fig — Data are shown as in S21 Fig. (TIF) [file ppat.1013392.s022.tif]

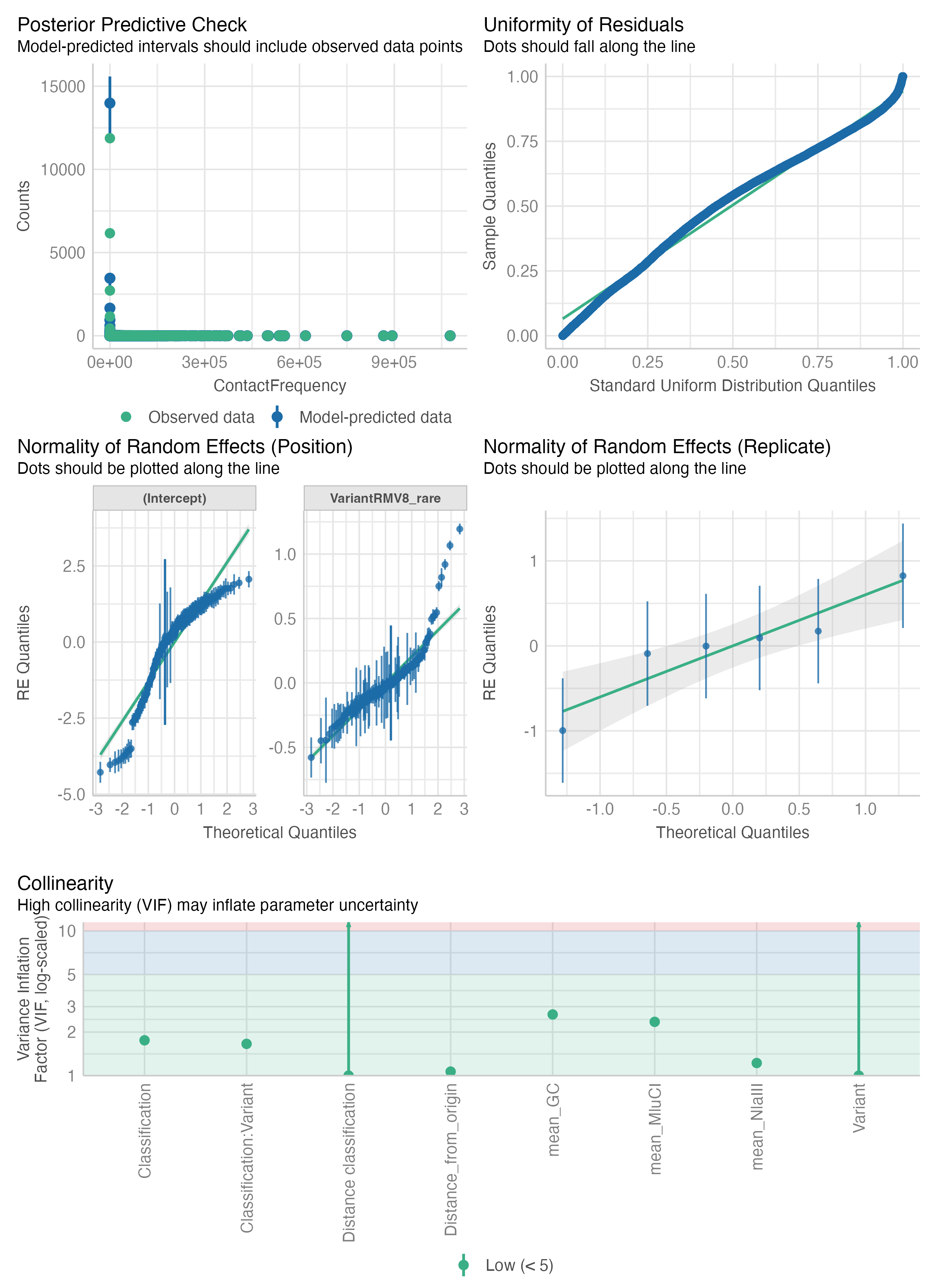

Supplement: S23 Fig — Data are shown as in S21 Fig. (TIF) [file ppat.1013392.s023.tif]

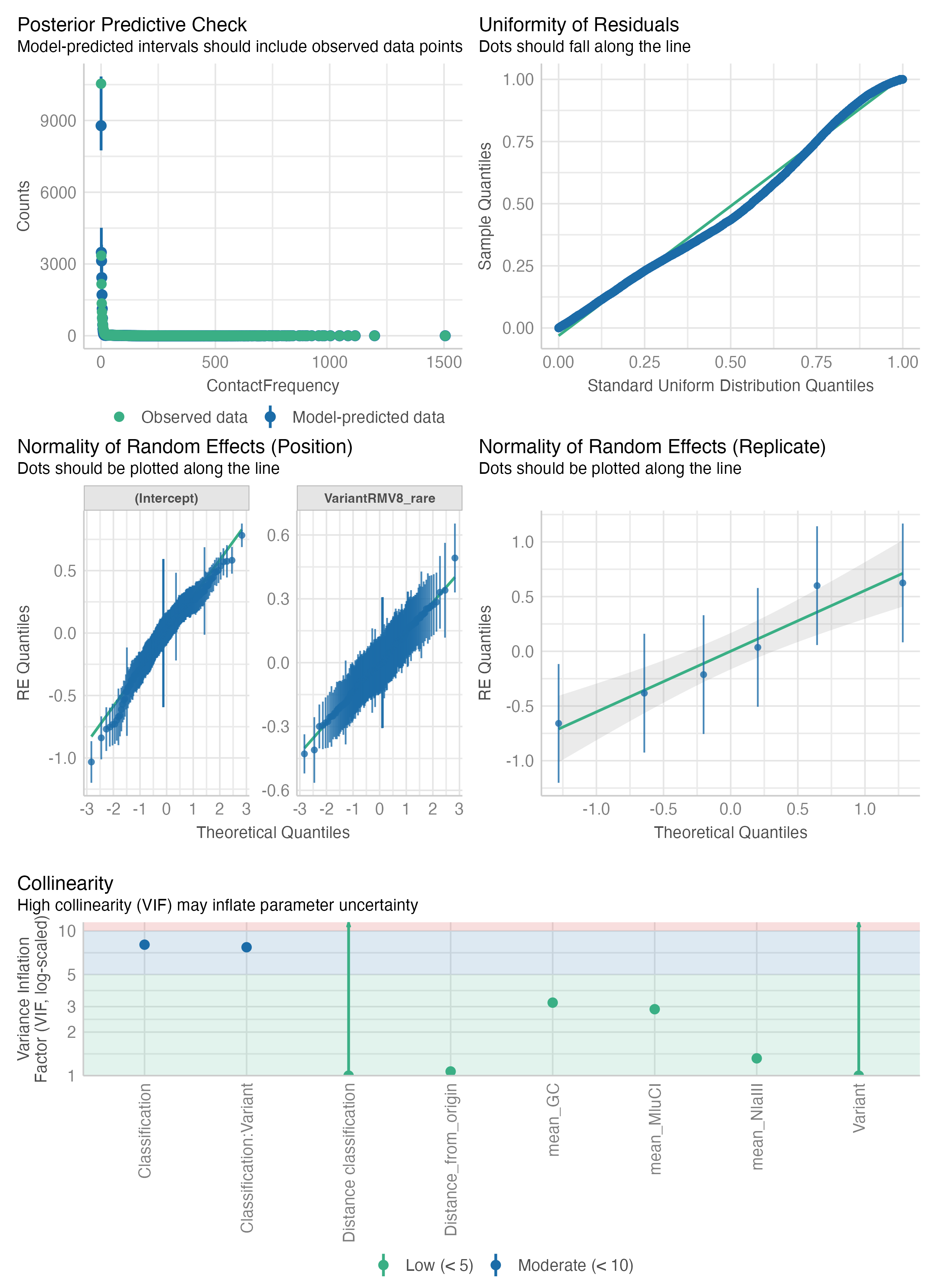

Supplement: S24 Fig — Data are shown as in S21 Fig. (TIF) [file ppat.1013392.s024.tif]
