# Supplementary material for: Pore-C sequencing identifies episome-driven chromosome conformation perturbations differentiating pneumococcal epigenetic variants
Source: PLoS Pathog. 2025 Aug 14;21(8):e1013392. doi: 10.1371/journal.ppat.1013392 (PMC12416852; doi:10.1371/journal.ppat.1013392)
Supplement: S1 Table — (DOCX) [file ppat.1013392.s026.docx]

| **Genotype** | **Description** | **Accession code of reference genome** |
| --- | --- | --- |
| RMV7_domi_ | The more common “domi” variant of RMV7 expressed a *Spn*IV allele that methylated the motif TGAN_7_TCC. | OV904788 |
| RMV7_rare_ | The less common “rare” variant of RMV7 expressed a *Spn*IV allele that methylated the motif TGAN_7_TATC. | - |
| RMV7_rare_ PRCI*_dnaN_*::Janus | RMV7_rare_ with PRCI integrated next to the gene *dnaN* replaced by a Janus cassette. | - |
| RMV8_domi_ | The more common “dominant” variant of RMV8 expressed a *Spn*IV allele that methylated the motif GATAN_6_RTC. | OX244288 |
| RMV8_rare_ | The less common “rare” variant of RMV8 expressed a *Spn*IV allele that methylated the motif GTAYN_6_TGA. | - |
| RMV8_rare_ PRCI*_malA_*::Janus | RMV8_rare_ with PRCI integrated next to *malA* replaced by a Janus cassette. | - |
| RMV8_rare_ ϕRMV8*::cat* | RMV8_rare_ with ϕRMV8 replaced by a chloramphenicol resistance marker. | - |
| RMV8_rare_ ϕRMV8*::cat*  PRCI*_malA_*::Janus | RMV8_rare_ with ϕRMV8 replaced by a chloramphenicol resistance marker and PRCI integrated next to the gene *malA* replaced by a Janus cassette. | - |
