# Supplementary material for: Pore-C sequencing identifies episome-driven chromosome conformation perturbations differentiating pneumococcal epigenetic variants
Source: PLoS Pathog. 2025 Aug 14;21(8):e1013392. doi: 10.1371/journal.ppat.1013392 (PMC12416852; doi:10.1371/journal.ppat.1013392)
Supplement: S2 Table — (DOCX) [file ppat.1013392.s027.docx]

| **Genotype** | **Replicate** | **Illumina Hi-C Accession Code** | **Nanopore Pore-C Run 1 Accession Code** | **Nanopore Pore-C Run 2 Accession Code** |
| --- | --- | --- | --- | --- |
| RMV7_domi_ | 1 | ERS17696384 | ERR13946717 | ERR13946729 |
| RMV7_domi_ | 2 | ERS17696392 | ERR13946718 | ERR13946730 |
| RMV7_domi_ | 3 | ERS17696382 | ERR13946719 | ERR13946731 |
| RMV7_rare_ | 1 | ERS17696383 | ERR13946720 | ERR13946732 |
| RMV7_rare_ | 2 | ERS17696385 | ERR13946721 | ERR13946733 |
| RMV7_rare_ | 3 | ERS17696386 | ERR13946722 | ERR13946734 |
| RMV8_domi_ | 1 | ERS17696387 | ERR13946723 | ERR13946735 |
| RMV8_domi_ | 2 | ERS17696388 | ERR13946724 | ERR13946736 |
| RMV8_domi_ | 3 | ERS17696389 | ERR13946725 | ERR13946737 |
| RMV8_rare_ | 1 | ERS17696390 | ERR13946726 | ERR13946738 |
| RMV8_rare_ | 2 | ERS17696391 | ERR13946727 | ERR13946739 |
| RMV8_rare_ | 3 | ERS17696393 | ERR13946728 | ERR13946740 |
