# Supplementary material for: Pore-C sequencing identifies episome-driven chromosome conformation perturbations differentiating pneumococcal epigenetic variants
Source: PLoS Pathog. 2025 Aug 14;21(8):e1013392. doi: 10.1371/journal.ppat.1013392 (PMC12416852; doi:10.1371/journal.ppat.1013392)
Supplement: S3 Table — (DOCX) [file ppat.1013392.s028.docx]

| **Genome** | ***Nla*III sites** | ***Mlu*CI sites** |
| --- | --- | --- |
| RMV7 | 6776 | 15897 |
| RMV8 | 6831 | 16425 |
| PRCI*_dnaN_* | 39 | 94 |
| PRCI*_malA_* | 25 | 90 |
