# Supplementary material for: Pore-C sequencing identifies episome-driven chromosome conformation perturbations differentiating pneumococcal epigenetic variants
Source: PLoS Pathog. 2025 Aug 14;21(8):e1013392. doi: 10.1371/journal.ppat.1013392 (PMC12416852; doi:10.1371/journal.ppat.1013392)
Supplement: S4 Table — (DOCX) [file ppat.1013392.s029.docx]

| **Name** | **Sequence** | **Function** |
| --- | --- | --- |
| RMV8_prophage_purA_chr_F (A)  RMV8_prophage_purA_int_F (C) | GGTCCTGGTCGTGAACAAAC  GCACCCTCCAAAAGCATTGA | Primers for assessing the topology of ϕRMV8. |
| RMV8_prophage_purA_chr_R (D)  RMV8_prophage_purA_int_R (B) | CTCAGCCTCTCTCAAAGCCT  AGTCCCCAAAAGCCTGAAAT | Primers for assessing the topology of ϕRMV8. |
| RMV8_A_chr_sugar_PRCI_F  RMV8_C_int_sugar_PRCI_F | ATTTATCCCCGCCACCCTTT  AGCCACTTATCCAAAGACAACA | Primers for assessing the topology of PRCI*_malA_*. |
| RMV8_B_int_sugar_PRCI_R  RMV8_D_chr_sugar_PRCI_R | TGAGGTGTGAAATGATGCCT  GGAAAATGGATATGGAAGCATAGGT | Primers for assessing the topology of PRCI*_malA_*. |
| RMV7_PRCI_uvrA_Chr_A_F  RMV7_PRCI_uvrA_int_C_F | GGTTCAAGCGTATCGATCTCT  GCGCGTGATATAAGGGTTAGG | Primers for assessing the topology of PRCI*_uvrA_*. |
| RMV7_PRCI_uvrA_int_B_R  RMV7_PRCI_uvrA_chr_D_R | TCTACGAAACTGCACTAAAAGC  GCGCCCCATGAATGACAATT | Primers for assessing the topology of PRCI*_uvrA_*. |
| RMV7_A_For_Chr  RMV7_C_For_int | AAAAGGCTAATCGTTGGGAAATT  GGCTCGGTCATGCAAAACTT | Primers for assessing the topology of PRCI*_dnaN_*. |
| RMV7_B_Rev_int  RMV7_D_Rev_Chr | AATATCAAGGGTTTAGGCGCT  CCAACGATACCTGCTGTCA | Primers for assessing the topology of PRCI*_dnaN_*. |
| malA_KO_UP_For  malA_KO_UP_Rev_ApaI  malA_KO_DOWN_For_BamHI malA_KO_DOWN_Rev | CAAAGCCTTACTTACCTTTACCTGAT  AAGGGCCCGAGTATAAAATGAAAAAGG  AAGGATCCCCTAGACTTGAAATAAAGC  TGAATAACACGGGTTACGGTTGAAG | Primers used to generate amplicons for the replacement of PRCI*_malA_* with a Janus cassette. |
| rpoA_Rev  rpoA_For | CACGAGCAGGTTCCACTTGA TGGTCGTGGATATGTACCTGC | Primers for the amplification of *rpoA* as a standard for qPCR experiments. |
| Janus_For  Janus_Rev | TTGGGCCCCCGTTTGATTTTTAATGGATAATGTG ATGGATCCCCTTTCCTTATGCTTTTGGACG | Primers for the amplification of the Janus cassette for mutant construction. |
| sugar_RMV8_qRT_For_int_tn2  sugar_RMV8_qRT_Rev_int_tn2 | GCCCGTGATATTATCGTGCC  GAATACGCAAACCAGTCGCT | Primers for the quantification of PRCI*_malA_* *int* gene expression |
| qRT_dnaC_RMV8_PRCI_For  qRT_dnaC_RMV8_PRCI_Rev | TACAACCATTTTGCCCGGAG  GGTATTTCTGAGCTTGCCCC | Primers for the quantification of PRCI*_malA_* *dnaC* gene expression |
